# Covalent adduction of endogenous and food-derived quinones to a protein: its biological significance

**DOI:** 10.3164/jcbn.18-26

**Published:** 2018-05-01

**Authors:** Yoji Kato, Naoko Suga

**Affiliations:** 1 Laboratory of Free Radical and Food Function, School of Human Science and Environment, University of Hyogo, 1-1-12 Shinzaike-honcho, Himeji, Hyogo 670-0092, Japan; 2 Research Institute of Food and Nutrition, University of Hyogo, 1-1-12 Shinzaike-honcho, Himeji, Hyogo 670-0092, Japan

**Keywords:** endogenous quinone, food-derived quinone, catechol, covalent modification, biological action

## Abstract

There are many chemically reactive compounds, including quinone, in living systems and also food. Even after the ingestion of food polyphenols, quinones derived from catechol moieties could form endogenously in the body. Dopaquinone, dopamine quinone, estrogen-derived quinones, tryptamine-4,5-dione, and ubiquinone are examples of an endogenous quinone. These indicate that quinone is ubiquitously formed or present in living systems and food. Quinones can induce a variety of hazardous effects and also could have beneficial physiological effects. This review focuses on the chemical reactivity of quinone toward a biomolecule and its biological action.

## Introduction

**Fig. 1:**
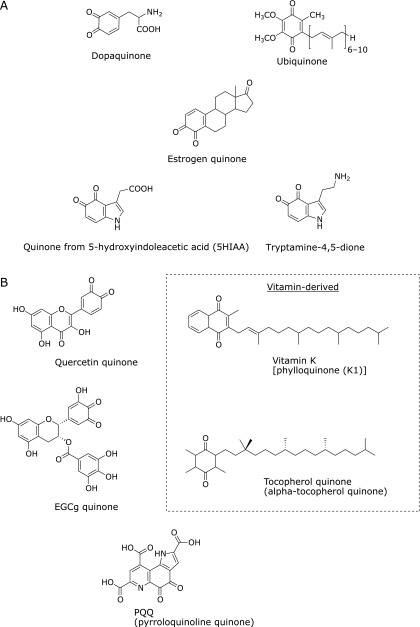
Chemical structures of endogenous and food-derived quinones (examples). (A) Endogenous quinones, (B) Food-derived quinones.

In living systems and food (i.e., substances derived from living systems), there are many chemically reactive compounds. Quinone is one of these and has a conjugated cyclic dione in its aromatic structure (Fig. 1). Depending on the position of the –C(=O)– group, this molecule is classified as *o*- or *p*-quinone. Quinone and its precursors are often taken into the body and can induce a variety of hazardous effects *in vivo*.^(1,2)^ Interestingly, some medicines have a quinone moiety in their structure, suggesting that quinone derivatives could have beneficial physiological effects.

We take in exogenous compounds from food. Some food (including medicinal plants, herbs, and spices) contains quinones, which are incorporated into the body through meals. Polyphenol oxidase produces indole-5,6-quinone, a brown pigment, with the ripening of fruit.^(3–5)^ Thymoquinone, the predominant constituent of *Nigella satica* volatile oil, exhibits favorable effects such as hepatoprotective, anti-inflammatory, antioxidant, and anticancer properties.^(6)^ Naphthoquinone derivatives (i.e., β-lapachone) are often found in plants and also exert some biological functions.^(7)^ Moreover, even after the ingestion of food-derived polyphenols (catechols), quinones can form endogenously in the gut or in organs/tissues.^(8–11)^ Some vitamins and related compounds also have or develop a quinone moiety. For example, vitamin K has a quinone moiety in its standard form, while tocopherol (vitamin E) forms tocopherol quinone through antioxidative action. Moreover, pyrroloquinoline quinone is known as a redox cofactor for an enzyme and could be a new vitamin,^(12)^ although this remains controversial.^(13)^

Living systems generate quinones endogenously, which include dopaquinone, dopamine quinone, or estrogen-derived quinones. Tryptamine-4,5-dione is formed by the oxidation of serotonin, 5-hydroxytryptamine.^(14,15)^ Ubiquinone is an endogenous quinone that plays a critical role as a component of the electron transport chain for aerobic respiration. Ubiquinone also serves as a lipid-soluble antioxidant in cellular membranes. In this way, quinone is ubiquitously formed or present in living systems and food.

**Fig. 2:**
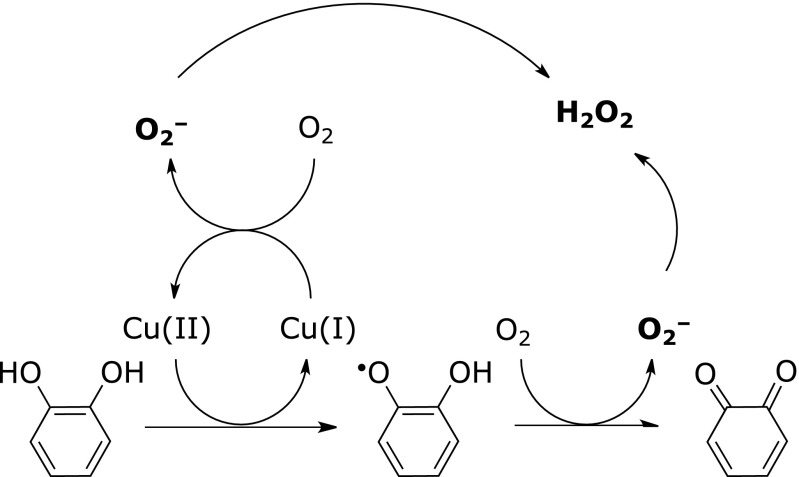
Generation of reactive oxygen species and quinone from catechol in the presence of transition metal ions and oxygen.

In cells, quinone has two major biologically relevant characteristics. One is as a reactive oxygen generator; quinone generates reactive oxygen species (ROS), such as superoxide and subsequently hydrogen peroxide. Anthraquinone (*p*-quinone) is a known example of this. A catechol derivative also generates superoxide in the presence of a transition metal, Cu^+^ (copper ion) or Fe^2+^ (ferrous ion), forming the corresponding *o*-quinone (Fig. 2). Two superoxide molecules transform into one hydrogen peroxide via dismutation. Menadione, a non-naturally occurring vitamin K derivative, also generates ROS in cells via two-electron detoxifying enzymes such as NAD(P)H:quinone oxidoreductase.^(16)^ The ability to generate ROS from quinone as a free or even conjugated form (as mentioned later) could account for some biological activity evoked by quinones.

**Fig. 3:**
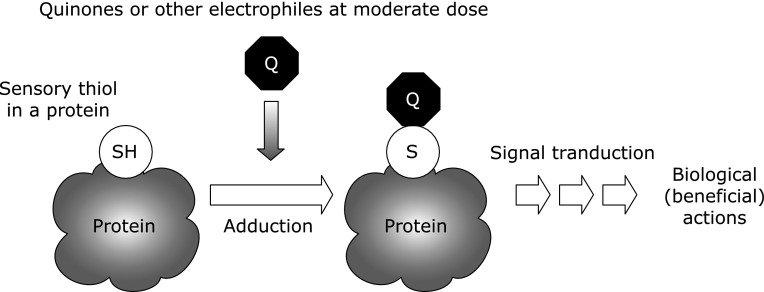
Sensory thiol is a critical target of quinone.

Another characteristic of quinones is their high reactivity toward biomolecules. For example, *o*-quinone readily reacts with free thiol moieties, followed by the formation of a covalent conjugate. In cells, thiols play important roles such as in cell signaling and as environmental sensors. The reaction of quinone with the sensory “thiol” could then initiate some biological responses (Fig. 3).

In this review, we focus on the chemical reaction and biological action of endogenously generated quinones, in particular tryptamine dione and its related quinones, along with food-derived quinones.

## Generation of Quinones and Reaction toward Biomolecules

Catechols are converted to corresponding quinones by the catalysis of transition metal ions or by enzymes such as peroxidase. Because many catechols are present in food, these food-derived catechols could have biological effects via the transformation of quinone, accompanied by the formation of an adduct with a biomolecule. When tea catechin, epigallocatechin gallate (EGCg), is autoxidized by molecular oxygen, the catechol in a gallate moiety is transformed into an EGCg quinone (Fig. 1) along with the accumulation of hydrogen peroxide derived from the generated superoxide.^(17)^ EGCg quinone or its analog reacts with a thiol in a protein and then the quinone returns to the catechol, dihydroxyl moiety.^(9,10,18)^ Tea has beneficial effects on human health and it has been speculated that these effects are caused by the components in tea. The quinone of EGCg could participate in some of the biological activities of tea;^(11)^ we discuss the mechanisms behind this in section 3 below. Flavonoid-derived quinones, such as quercetin quinone, might be key players in the induction of cell protection against oxidative stress.^(8)^ Notably, some flavonoids or anthocyanins are unstable under physiological conditions or catabolized by gut microflora. 3,4-Dihydroxyphenylacetic acid, a breakdown product from quercetin glycosides, could be absorbed into the circulation and show biological activities through electrophilic adduction of the corresponding quinone to biomolecules.^(19)^

**Fig. 4:**
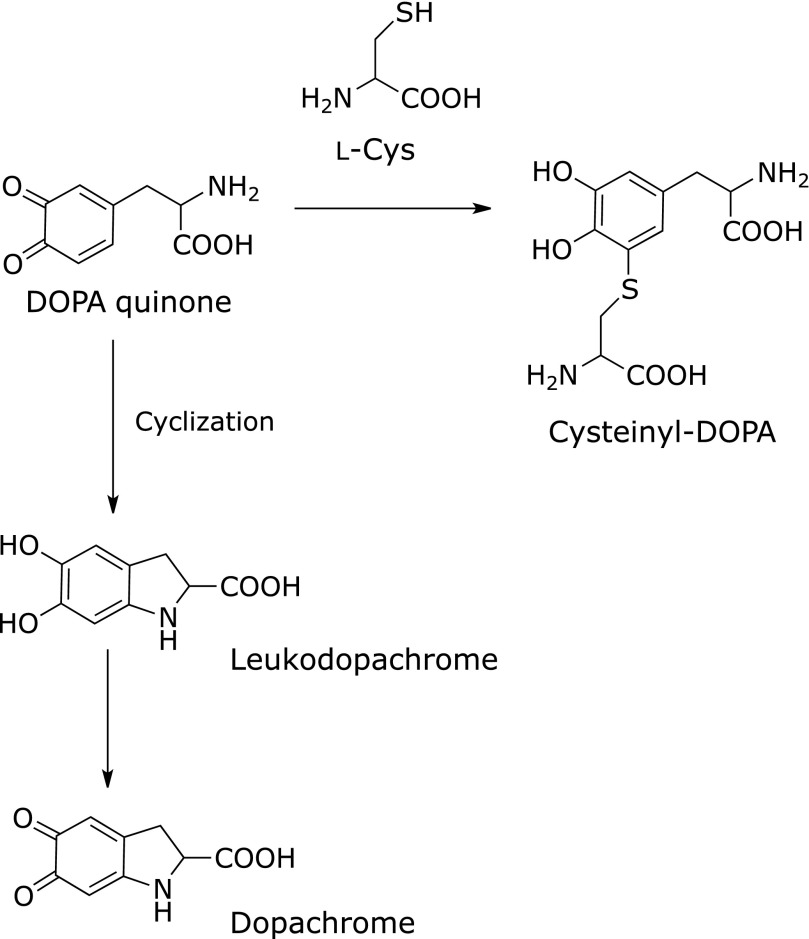
Reactivity of *o*-quinone. Adduction to a thiol moiety or self-cyclization.

Dopaquinone is enzymatically generated from l-dihydroxylphenylalanine (DOPA), which originates from l-tyrosine.^(20)^ Dopaquinone is further converted into dopachrome by self-cyclization or forms 5-*S*-cysteinyl-dopa by conjugation with l-cysteine (Fig. 4). Subsequently, melanins (eumelanin, pheomelanin, and neuromelanin) are formed through multiple complicated steps.^(20)^ Dopaquinone covalently adducts to a thiol, dihydrolipoic acid,^(21)^ and also inactivates tryptophan hydroxylase accompanying the formation of quinoprotein.^(22)^ Along with dopaquinone, dopamine quinone is also a possible key factor in neurodegenerative diseases. Proteomic analyses have shown that a subset of proteins were decreased or modified by exposure of the quinone and that dopamine quinone conjugated to glutathione peroxidase 4 *in vitro*.^(23–25)^ The cysteinyl adduct of dopamine quinone on a protein generates ROS in the presence of copper ion.^(26)^

Estrogens are important regulators of female reproductive functions. However, excessive estrogen can initiate carcinogenesis. When estrogens [estrone (E1) and estradiol (E2)] undergo oxidative metabolism by specific cytochrome P450, catechol estrogens, 2-hydroxyestrone (2-OHE1), 4-hydroxyestrone (4-OHE1), 2-hydroxyestradiol (2-OHE2), and 4-hydroxyestradiol (4-OHE2), are formed. These are further oxidized to quinones. Because the quinones, and in particular estradiol-3,4-quinone (Fig. 1),^(27)^ react with DNA, the adduction could be a cause of breast cancer.^(28)^ In addition, quinones derived from catechol estrogens also form covalent bonds with cysteine (and in part histidine/lysine) residues in neuroglobin and serum proteins.^(29,30)^

**Fig. 5:**
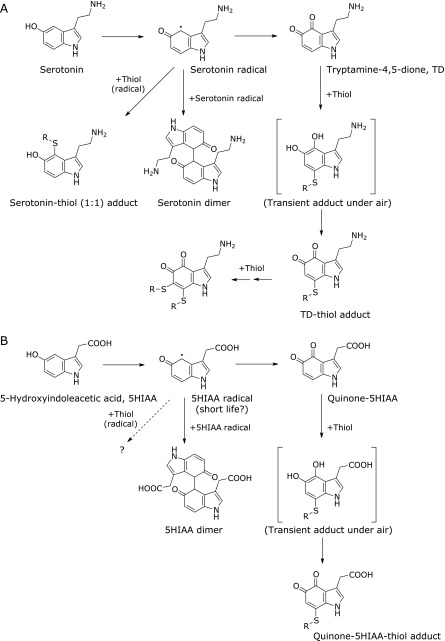
Schematic diagram for the generation and reaction of serotonin- and its metabolite 5-hydroxyindoleacetic acid (5HIAA)-derived quinones by myeloperoxidase in the presence of hydrogen peroxide. (A) serotonin, (B) 5-HIAA.

Tryptamine dione is generated from the oxidation of serotonin by superoxide or by peroxidases (Fig. 1).^(14,15)^ It has been reported that mercury-treated zebrafish brain contains tryptamine dione,^(31)^ which is a potential neurotoxin.^(32)^ Pioneering studies concerning tryptamine dione were conducted in the 1960s by Dryhurst’s group, from which the synthesis, stability, and reactivity of tryptamine dione were reported.^(14,33–37)^ The generator of tryptamine dione *in vivo* is not clearly known, but serotonin and other indoles are good substrates for peroxidases such as myeloperoxidase.^(38,39)^ When myeloperoxidase oxidizes serotonin into serotonin radicals, both serotonin dimer and tryptamine dione are then formed.^(15,40)^ The tryptamine dione has high reactivity toward a thiol and then forms an adduct. After this adduction, the formed catechol moiety is further oxidized spontaneously and the quinone is then regenerated rapidly.^(34)^ The formed thiol adduct could be conjugated with one more thiol, accompanied by the formation of two thiol-adducted conjugates (Fig. 5A).^(40)^ In addition, a serotonin-thiol adduct was also observed. The quinone-thiol adduct with free l-cysteine or glutathione is unstable and further decomposed,^(34)^ indicating that low-molecular-weight thiol conjugates would not be stable markers for the *in vivo* generation of the quinone. The specific thiol residues in glyceraldehyde-3-phosphate dehydrogenase were modified by tryptamine dione.^(40)^ As shown below, the quinone-modified protein was immunochemically detected on a membrane or fixed tissue, indicating that adducted quinone on a protein is relatively stable.^(41,42)^

Serotonin is metabolized into 5-hydroxyindoleacetic acid (5HIAA) by the enzymatic actions of monoamine oxidases and aldehyde dehydrogenase *in vivo*. During study on serotonin oxidation by myeloperoxidase, our group has found that 5HIAA is effectively oxidized by the enzyme and quinone of 5HIAA is then formed (Fig. 1).^(41)^ The confirmation of the novel quinone was performed by chemical trapping of the quinone by *o*-phenylenediamine to form the corresponding phenazine derivative.^(43,44)^ In the presence of *N*-acetyl-l-cysteine, the quinone of 5HIAA adducted to the free thiol, but 5HIAA-*N*-acetyl-l-cysteine adduct was not confirmed, probably because of the short life-time of 5HIAA radicals compared with that of serotonin radicals (Fig. 5B).^(40,41)^ Monoclonal antibodies to quinone-5HIAA-modified protein or tryptamine dione-modified protein were also developed and applied to the immunohistochemical staining of human atherosclerotic plaque. The results revealed positive staining by both antibodies at the loci.^(41)^ These results suggest (1) the *in vivo* presence of quinones from both serotonin and 5HIAA, and (2) their adduction toward biomolecules *in vivo*. There are only a few reports on the detection of tryptamine dione *in vivo* and no reports on quinone from 5HIAA.^(31,45)^ Chemical confirmation of the *in vivo* generation of the quinones should be performed in the future. The biological significance of quinones in atherosclerotic lesions also remains to be explored.

As mentioned above, quinone compounds can form covalent adducts with thiol moieties, a reaction that is generally considered to be irreversible. However, Miura *et al.*^(46)^ reported a unique restoring reaction from quinone-adducted protein through glutathione-dependent *S*-transarylation. We also found that, once conjugated, tryptamine dione moves to a thiol residue in another protein, which is disrupted by *N*-acetyl-l-cysteine supplementation (unpublished observation). Endogenous quinone might transfer from the first target molecule to the second target, leading to modulation of signal transduction in a cell.

In this section, we describe how to analyze free quinone and how to visualize quinone adducts in a protein. Free quinone itself tends to be unstable and to have high reactivity toward biomolecules. Chemical trapping for *o*-quinone by a diamine moiety can be applied to detect and quantify free *o*-quinone, as mentioned previously.^(41,43,44)^ Quinone staining by incubation with nitroblue tetrazolium in alkaline glycinate buffer has been developed and applied for the detection of quinoproteins (quinone-adducted proteins).^(47,48)^ The use of a biotinylated analog combined with (strepto)avidin-labeled enzyme/beads or an analog having an azide moiety for click chemistry is another alternative.^(9,19,40)^ Specific antibodies to quinone or quinone adduct have also been used for immunochemical detection.^(42,49,50)^

## Biological Effect of Quinone on Cells/Tissues

The covalent modification of a sensory thiol protein by a highly reactive electrophilic molecule including quinone can have certain biological effects (Fig. 3).^(51,52)^ Hence, endogenous quinones have a biphasic role in maintaining or disrupting biological functions. Some studies have analyzed the effects of various quinones on cells, examples of which are presented below.

The dietary intake of fruit and vegetables is expected to reduce the risk of cardiovascular diseases. Heme oxygenase-1 (HO-1), which has a critical role in vascular protection,^(53,54)^ is upregulated by the treatment of cells or animals with some flavonoids (e.g., quercetin).^(55–58)^ A recent report described that quinone derivative of caffeic acid but not its dihydroxy derivative induces the expression of HO-1 in human aortic endothelial cells.^(8)^ Moreover, the quinone protects *ex vivo* arteries from mouse against hypochlorite-induced dysfunction. These actions via HO-1 might explain in part the beneficial effect of the ingestion of fruit and vegetables on cardiovascular function.^(8)^

The green tea polyphenol EGCg is one of the molecules potentially behind the health benefits of drinking tea. Tachibana *et al.*^(59)^ reported the EGCg binds to the 67-kDa laminin receptor. After the binding of EGCg to this receptor, the signaling starts at eEF1A and MYPT1 phosphorylation, leading to the activation of myosin phosphatase.^(60)^ Then, the receptor-based interaction leads to the expression of anticancer activity. On the other hand, the ingestion of EGCg might induce biological effects via the conversion of EGCg to the corresponding quinone, which could covalently react with a biomolecule in a cell. A recent study showed that EGCg directly bound to cellular proteins in AZ521 human gastric cancer cells through autoxidation.^(61)^ Among the modified cellular proteins, the DEAD-box RNA helicase p68 was identified as a novel EGCg-binding target. Because the DEAD-box is often overexpressed in a variety of tumor cells and plays an important role in cancer development and progression, the adduction to p68 could play an important role in cancer chemoprevention by tea. The paper also shows that EGCg inhibits AZ521 cell proliferation by preventing β-catenin oncogenic signaling through the proteasomal degradation of p68. Meanwhile, a study revealed that EGCg inhibited angiotensin-converting enzyme (ACE) activity through oxidative conversion into quinone and successive covalent binding of the quinone to ACE.^(11)^ Furthermore, the paper revealed that EGCg is a non-competitive inhibitor of ACE and EGCg binds to a specific site but not the active site of ACE.

Dopamine quinone and dopaquinone are representative endogenous quinones and have been well researched.^(62,63)^ The quinones are possible elicitors of Parkinson’s disease by modifying neuronal proteins and successively affecting their functions.^(63)^ For example, dopamine quinone covalently adducts with Parkin (E3 ubiquitin ligase) and then inhibits its ligase activity.^(64)^ α-Synuclein is one target of the quinones, the adduction of which induces the aggregation of α-synuclein,^(65)^ which is a possible major cause of Parkinson’s disease. Moreover, dopamine suppressed NOx accumulation in the murine microglial cell line BV-2 evoked by lipopolysaccharide; this might have been caused by the binding of dopamine quinone with a cysteine residue of signaling molecules, such as c-Jun N-terminal kinase.^(66)^

As mentioned above, specific metabolites of endogenous estrogens, the catechol estrogen-3,4-quinones, react with DNA and contribute to gynecological cancers.^(67)^ Besides, a new effect of estrogen quinones has been noted, namely, the covalent modification of insulin by estrogen quinones reduces glucose uptake by affecting insulin signaling via insulin receptor substrate 1 phosphorylation in MCF-7 cells.^(68)^

As shown earlier in this paper, tryptamine dione is a possible endogenous quinone. By treating human SH-SY5Y neuroblastoma cells with tryptamine dione, some cellular proteins were found to be specifically and also non-specifically modified by the quinone.^(42)^ Among these, some cytoskeletal proteins, such as α, β-tubulins, vimentin, β-actin, and neurofilament-L, were identified as tryptamine dione-modified proteins. Because these proteins are abundant in cells, it is suggested that non-specific modification occurs there. Such non-specific binding could cause “proteo-stress” followed by the induction of heat shock factor 1 and successive biological actions.^(69)^ Of the tryptamine dione-modified proteins, tubulins have critical functions in mitosis, cell motility, and intracellular transport. When tubulins were exposed to tryptamine dione, self-polymerization was initially enhanced, but later inhibited.^(42)^ This indicates that the modification of proteins by a quinone could modulate their function.

**Fig. 6:**
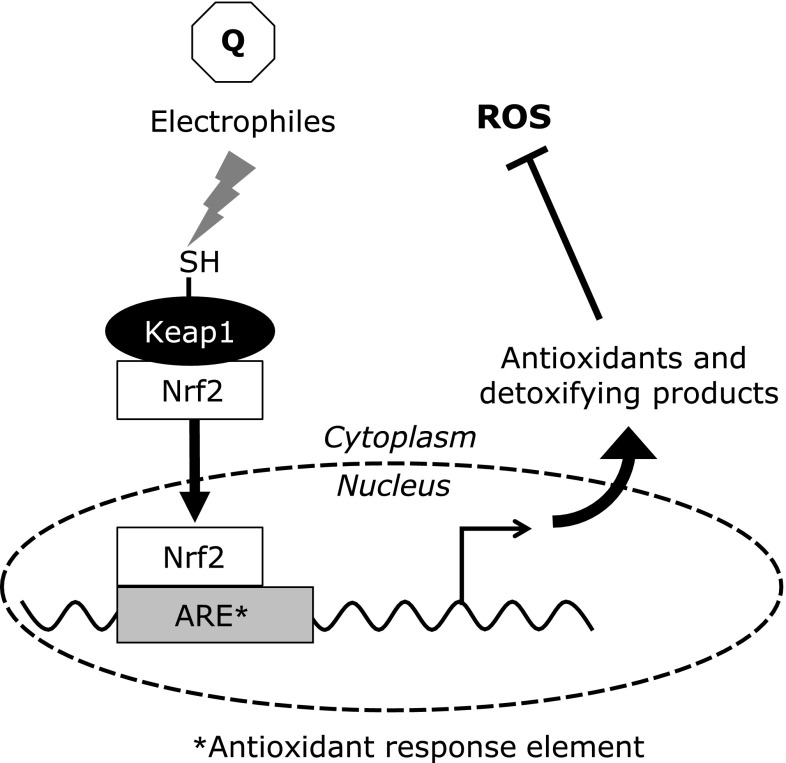
Adaptive response initiated by electrophiles including quinone. After initial priming (adduction to thiol residue of Keap1), Nrf2 binds to antioxidant response element and stimulates the production of some cytoprotective proteins. Then, cells are ready to protect against reactive oxygen species.

It has often been asserted that toxic compounds have double effects on cells, like Jekyll and Hyde. Tryptamine dione also acts as a kind of double-edged sword.^(70)^ The viability of SH-SY5Y cells was significantly decreased by exposure to tryptamine dione at a high dose, with increasing intracellular ROS. Conversely, treatment with a moderate dose of tryptamine dione increased the viability of cells. It has been suggested that mild stress, such as low levels of phytochemicals (toxins) in fruit and vegetables, has beneficial effects by activating adaptive cellular stress response pathways.^(71)^ This implies that the treatment of cells with tryptamine dione or other reactive quinones at moderate concentrations may increase the self-defense capacity of cells against oxidative stress. In fact, pretreatment of SH-SY5Y cells with tryptamine dione suppressed cell death and the generation of intracellular ROS evoked by the subsequent addition of hydrogen peroxide. The expression of phase-II antioxidant enzymes, NAD(P)H: quinone oxidoreductase 1 (NQO1) and HO-1, was upregulated by tryptamine dione at a moderate concentration. Nuclear factor erythroid 2-related factor 2 (Nrf2), the key/master regulator of these enzymes, was translocated from the cytosol to the nucleus by treatment with tryptamine dione. Nrf2 is regulated by Kelch-like ECH-associated protein 1 (Keap1), which has reactive thiol residues that act as a sensor of electrophiles and/or oxidants (Fig. 6).^(72)^

Similarly, some electrophiles including estrogen quinones, 2-OHE2, 4-OHE1, and 4-OHE2 quinones could covalently adduct with specific Keap1 thiols and upregulate Nrf2-dependent gene expression via antioxidant response element.^(1,73)^ Therefore, a quinone may adduct with the thiol residue of Keap1, leading to adaptive expression of the defense system via some cytoprotective proteins (HO-1, NQO1). This provides a status of resistance to subsequent oxidative stress. These stressor molecules (quinones), which cause mild stress, could be considered as a “primer” of cellular response. In this way, the cellular responses might be triggered by covalent binding of the quinone with cellular proteins, at least in part. Quinones could have both beneficial and adverse effects on cells, depending on the amount and site of formation.

## Conclusion

In this review, we have focused on the biological actions of quinones derived from endogenous and exogenous sources. Generally, both food-derived and endogenous reactive chemicals may share pathways through which they exert their biological functions, which are triggered by a reaction of the chemicals with sensory thiols in cells/tissues.^(74)^ Although the high reactivity of quinones toward biomolecules sometimes has harmful effects, they mostly have beneficial functions in living systems if the dose is in the moderate range.
